# Superior Approach of Recurrent Laryngeal Nerve: Review of the Literature

**DOI:** 10.1155/2019/5671816

**Published:** 2019-12-18

**Authors:** Koné Fatogoma Issa, Dao Sidiki, Cissé Naouma, Diarra Kassim, Konaté N'Faly, Samaké Djibril, Tafo Neuilly, Haidara Abdoul Wahab, Guindo Boubacary, Soumaoro Siaka, Singaré Kadidiatou, Timbo Samba Karim, Kéita Mohamed Amadou

**Affiliations:** ^1^University Hospital of Gabriel Touré, Bamako, Mali; ^2^Health Center of Common Reference I, University Hospital of Gabriel Touré, Bamako, Mali; ^3^Health Center of Common Reference V, University Hospital of Gabriel Touré, Bamako, Mali; ^4^Health Center of Common Reference III, University Hospital of Gabriel Touré, Bamako, Mali; ^5^University Hospital of Luxembourg, Luxembourg, Luxembourg

## Abstract

The identification and dissection of the recurrent laryngeal nerve is essential to guarantee its anatomical and functional integrity. The superior approach of the recurrent nerve is a reliable surgical alternative. Various indications are recognized with a reliable landmark. This is the entry point into the larynx under the inferior horn of the thyroid cartilage. The limits of this technique, namely, the extralaryngeal divisions and the hemorrhages encountered at the point of entry of the larynx are a source of morbidity of the recurrent laryngeal nerve. A careful dissection, respect for the surgical steps, and the surgeon's experience are guarantees of a good result. We wanted through a review of the literature and our experience in the superior approach to discuss surgical indications, to identify landmarks at the point of entry of the larynx, to determine the limits of this approach, and to take precautions to mitigate the risk of recurrent laryngeal nerve injury.

## 1. Introduction

Surgery of the thyroid gland requires perfect anatomical knowledge of the neck and its anatomical variations [1]. The identification of the recurrent nerve during thyroid surgery is the gold standard in the prevention of nerve damage and the reduction of recurrent paralysis [1, 2]. The different approaches of the recurrent laryngeal nerve depend on the indications and on the surgeon's habit. Several approaches exist such as the superior approach, the lateral approach, and the inferior approach [3]. Lubrano in his review emphasized the inferior approach [4]. Besides this, the superior approach is defended by some authors but with a restriction on these indications [5]. Wang showed interest on the approach of the recurrent nerve in the inferior horn of the thyroid cartilage in 1975 [6]. Multiple works have followed the work of Guerrier, Page, and Butskiy [1, 3, 5]. Our center focused on the superior approach of the recurrent laryngeal nerve in preventing the recurrence of giant goiters [7]. All these studies have focused on the fidelity of the recurrent laryngeal nerve at the point of entry into the larynx with the need for mastery of its anatomical environment [3, 5, 7]. The anatomical variations that the nerve develops at the point of entry into the larynx and the haemorrhage constitute a major risk of nerve injury. We wanted, through our experience and a review of the literature, to discuss the indications, the reliability of the landmarks, and the precautions to take to avoid a possible recurrence risk during the upper approach of the recurrent laryngeal nerve.

## 2. Indications of the Superior Approach

Different studies show indications for the superior approach. These indications prove the limitation to the recourse of the other approaches. According to Page, the indications concerned cervicothoracic goiters [5]. Guerrier reported the goblin goiters with at least one lobe creeping into the cervicothoracic defile and reaching the level of the aortic arch [8]. There were also large goiters with significant inferior extension, causing problems of externalization [8]. Secondary thyroidectomies for recurrence of multiheteronodular goiters occurring on previously operated thyroid lodges have also been included [8]. According to Guerrier, there is no contraindication to the realization of this technique in goiters with upper development [8]. This joins the study of Butskiy, who systematized the superior approach of the recurrent laryngeal nerve [3]. In an experiment of 131 dissected recurrent nerves, the dissection was systematic. In situations where the Berry ligament is difficult to approach as a precaution and depending on the size of the gland, we approach either the lateral or the inferior level. These changes are the main recourse and are exceptional. In our center, we resorted to this in 3% of the cases. Nevertheless, the study of Guerrier has preserved it for difficult cases where the realization of the classical technique proves difficult [8]. All authors have enrolled in the reliability and consistency of the recurrent laryngeal nerve at the point of entry into the larynx [3, 5–9]. According to the literature review, the nonrecurrent laryngeal nerve should be sought when the recurrent laryngeal nerve is not visualized in its usual place according to conventional scoring methods [4]. Few methods focus on the identification of the nonrecurrent laryngeal nerve. This approach can extend to the nonrecurrent laryngeal nerve especially when the diagnosis is made preoperatively based on indirect signs such as the presence of the artery lusoria. Apart from these cases, we have found the nerve faithful to the point of entry.

## 3. Technique of Dissection

**Figure 1:**
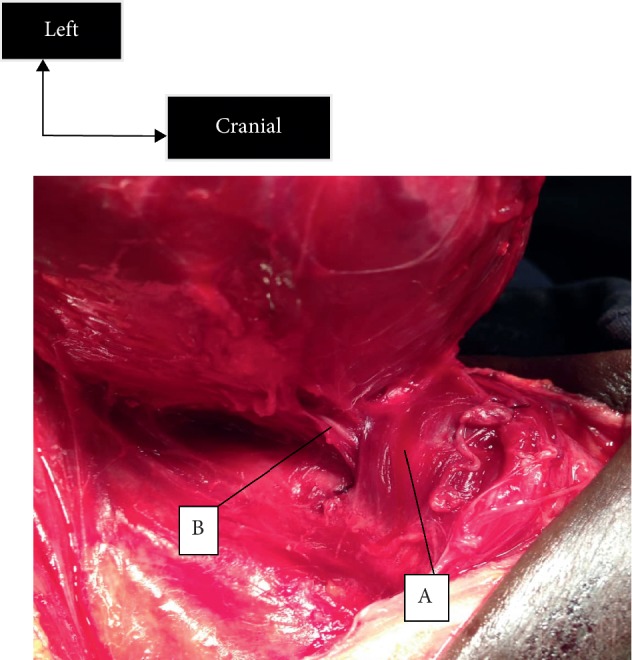
The cricopharyngeal muscle and the left recurrent laryngeal nerve at the point of entry into the larynx. A, cricopharyngeal muscle; B, recurrent laryngeal nerve.

**Figure 2:**
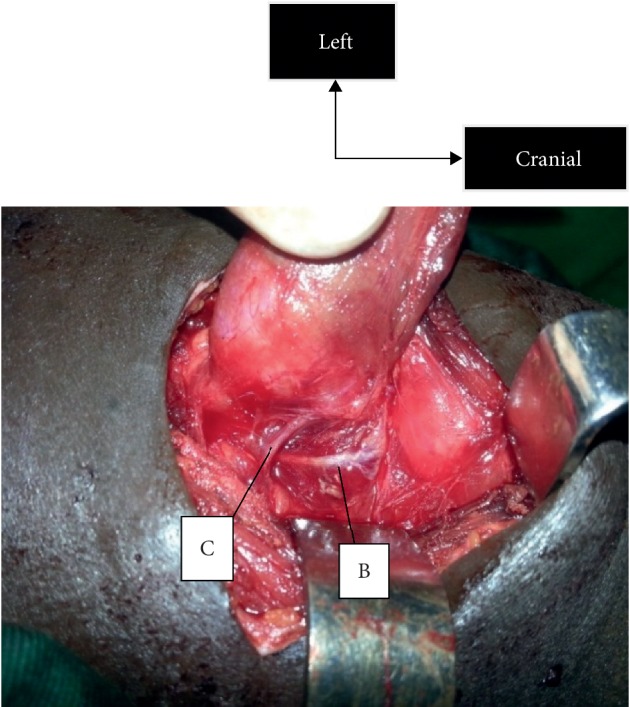
Dissection of the left recurrent laryngeal nerve until it meets the inferior thyroid artery. B, recurrent laryngeal nerve; C, inferior thyroid artery.

**Figure 3:**
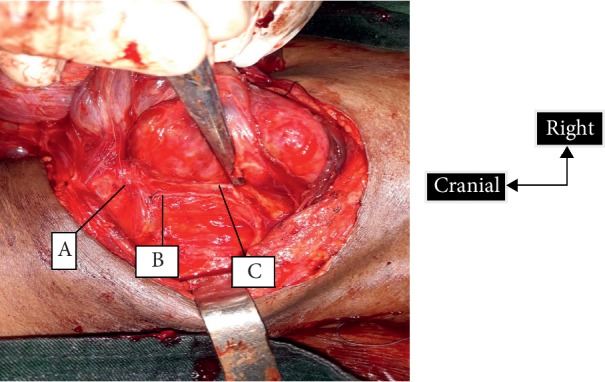
The relationship between the right recurrent laryngeal nerve and the posterior branch of the inferior thyroid artery. A, cricopharyngeal muscle; B, recurrent laryngeal nerve; C, posterior branch of the inferior thyroid artery.

According to the different approaches, the superior approach obeys the principle of thyroid surgery in terms of the patient's facility, operative position, and cervical incision. The incision extends from 6 to 10 cm of the Kocher type according to the volume of the goiter [5]. In our experience, the mini-incision less than 6 cm was performed in some patients with a small-sized gland. According to the recommendations, the size of the incision does not dispense with the identification and dissection of the recurrent laryngeal nerve. After exposure of the gland after the medial aponeurotomy, some authors prefer the first isthmic section. This section is not required. Ligation of the vessels of the superior pole, the artery and the superior thyroid veins, is required. Thus, the gland is detached through the avascular zone between the cricothyroid muscle and the superior lobe. The superior thyroid lobe is pulled down and inwards. The preservation of the parathyroid is important. Identification at the point of entry requires mastery of its anatomy. The authors recommend the monitoring of the nerve to facilitate its identification [5, 8]. The monitoring of the recurrent laryngeal nerve is necessary, but we do not have this tool at the moment. The analysis of the various studies reporting this technique has highlighted the various references (Figures 1–3).

### 3.1. Landmarks

- *Exposure of the Cricopharyngeal Muscle* (Figures 1 and 2). Different sizes of the incision, if it is made for thyroidectomy, give access to the ligation of the vessels of the superior and inferior poles, and tracking by the superior approach is possible because the exposure of the cricopharyngeal muscle is possible. The exposure of this muscle is an essential element in the realization of this technique. The cricopharyngeal muscle is carefully exposed through an avascular zone between the superior pole and the thyroid cartilage. The lower edge arranged in a sling around the thyroid cartilage is opposite to the point of penetration [8]. Of the 115 dissected nerves in the Warrior series, the set had a reliable entry point under the cricopharyngeal as well as in the series in [5, 8]. In the series by Nyekia, 62 recurrent laryngeal nerves had a reliable point of entry [9].
- *The Inferior Horn of the Thyroid Cartilage.* The interest of palpation of the inferior horn of the thyroid cartilage was described by Wang in 1975 [6]. The inferior horn of the thyroid cartilage is palpated to testify the depth of the recurrent laryngeal nerve [8], in cases where palpation becomes difficult and reference is made to the posterior projection of the cricothyroid membrane that corresponds to the inferior horn of the thyroid cartilage [8]. The advantage of this approach is that the position of the recurrent laryngeal nerve with respect to the cricothyroid junction is the same regardless of the pathology of the thyroid and congenital variations [3]. It is considered to be the most fixed landmark of the recurrent laryngeal nerve to the point of entry into the larynx [3]. All 62 dissected nerves entered the larynx behind the inferior horn of the thyroid cartilage [3]. This marker is 0.8 cm ± 4 mm below and behind the inferior horn of the thyroid cartilage and is easily palpable [10]. Uen has concluded on a series of 120 dissected nerves that the inferior horn of the thyroid cartilage is a reliable landmark of identification of the recurrent laryngeal nerve [10].
- *The Berry Ligament.* According to Sasou's study of 25 dissections, the distance between the recurrent laryngeal nerve and the Berry ligament ranged from 1 to 7 mm with an average distance of 3.1 mm [11]. It is important to determine the relationship between the recurrent laryngeal nerve and the Berry ligament to prevent nerve damage during thyroid surgery; nerve damage most often occurs in this area during total thyroidectomy [11]. Nyekia reported that 58 out of 62 dissected nerves were posterolateral to the Gruber ligament or 93.6% of the series, and the other 4 (6.4%) passed between the fibers of this ligament [9]. Uen noted that the right recurrent laryngeal nerve in 99.6% was within 3 mm of the Berry ligament on the right and in 86.6% on the left, whereas in 0.4% on the right and in 14.6% on the left, the right recurrent laryngeal nerve was outside the Berry ligament [10]. This ligament directs towards the nerve. In situations where the Berry ligament is difficult to approach as a precaution and depending on the size of the gland, we approach either the lateral or the inferior level. These changes are the main recourse and are exceptional. In our center, we resorted to this in 3% of the cases.
- *The Branch of the Posterior Thyroid Artery* (Figure 3). In 112 cases (84.2%), the posterior division of the inferior thyroid artery accompanying the recurrent laryngeal nerve was observed in the Guerrier series [8]. As for the lesion of the posterior branch of the inferior thyroid artery, we recorded 20.55% of minimal lesions of the posterior branch. Most often hemostasis is obtained by fine compression on the site. This relationship between the posterior branch of the inferior thyroid artery and the recurrent nerve was found in Koné's study [7]. The main sources of haemorrhage emanate from this posterior branch of the inferior thyroid artery. Nyekia found that, in 82.25%, an arteriolar haemorrhage frequently encountered at the point of entry into the larynx during dissection [9]. Butskiy recommends judicious dissection and control of the terminal branch of the inferior thyroid artery to prevent haemorrhage that may make visualization of the nerve difficult [3]. In 20.3%, Guerrier noticed bleeding near the area of discovery of the nerve [8]. The proximity between this branch and the nerve is an alert for the surgeon when the branch is visualized before the nerve.

### 3.2. Conduct of Dissection

The recurrent laryngeal nerve search is carried out in a triangle bordered at the bottom by the lower edge of the muscle and at the top of the thyroid lobe. At this level, the chisel is outlawed given its largesse and the sharp end. We conduct the research with a soft-tip dissecting forceps. Recurrent laryngeal nerve research is done with the nerve in mind that the nerve is lateral to the trachea and in the oesotracheal angle to the left. The dissection should be done at the level of the lateral face of the trachea and at the level of the superficial part of the nerve. Low dissection below the trachea exposes the nerve to recurrent injury in the presence of extralaryngeal division. The discovery of a small branch should prompt the surgeon to conduct his dissection in a careful way to discover a second branch [4]. No coagulation should be performed at this level. The control of bleeding is important by visualizing the posterior branch of the inferior thyroid artery. In case of injury, this artery must be carried out a fine compression by means of a wet compression before the discovery of the nerve if not to expose and ligate the vessel which bleeds. A real puncture-dissection is performed without real contact with the nerve. The dissection is conducted at the level of the upper part of the nerve without removing it from its lateral and posterior environment until it meets the lower thyroid artery. Thus, we proceed according to the impairment to loboisthmectomy or total thyroidectomy. In situations where total thyroidectomy is indicated, we take care to spare the parathyroids which are four in number. In our experience, we noted 0.73% of cases of transient unilateral recurrent palsy and no case of definitive hypoparathyroidism.

## 4. Traps Encountered during the Superior Approach

**Figure 4:**
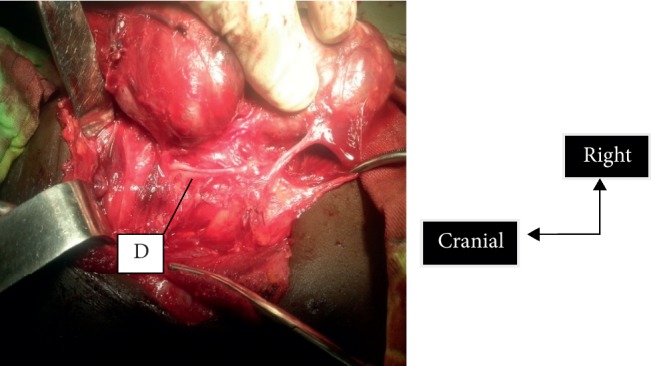
Trifurcation of the recurrent laryngeal nerve at the point of entry into the larynx. D, trifurcation of the recurrent laryngeal nerve.

**Figure 5:**
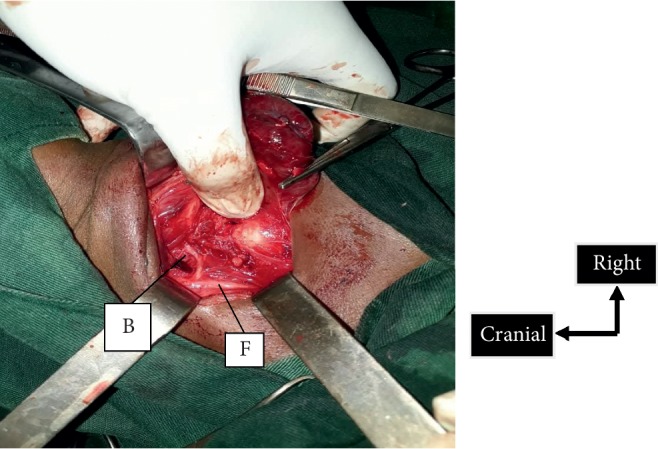
Recurrent nonrecurrent laryngeal nerve type 1. B, recurrent nonrecurrent laryngeal nerve; F, vagus nerve.

The entry of the recurrent nerve may be the site of multiple variations (Figure 4). In the Nyekia series, 9.7% of extralaryngeal divisions of the recurrent laryngeal nerve was found in 62 dissected nerves [9]. Extralaryngeal divisions are encountered; the discovery of a small branch is a source of morbidity. According to Lubrano, the discovery of a small branch is a warning sign for a surgeon to look for a second branch [4]. It is important to defer coagulation in this region. The posterior branch of the main lower thyroid artery causes haemorrhage at the point of entry. It is confusing with the recurrent laryngeal nerve as it is visualized before the recurrent laryngeal nerve. A crucial notion atrial flutter and its direction towards the arterial trunk make it possible to raise the margin of diagnostic error with the nerve. Visualization of the recurrent laryngeal nerve becomes difficult, when it is intermingled in Berry's ligaments. Careful dissection allows detachment of the ligament from the entrance area. At this level, the dissection should be conducted with a soft-end dissecting forceps. Research begins at the top, cricopharyngeal muscle and trachea. This makes it possible to detach the gland from the trachea and preserve the anterior branch of the recurrent laryngeal nerve in case of extralaryngeal division. In cases of multinodular goiter, the nodule develops at the bottom of the recurrent laryngeal nerve. The nerve is pushed upwards and becomes superficial, most often taking it as one of the branches of the superior thyroid artery. When it comes to the nonrecurrent recurrent nerve (Figure 5), it is one of the effective methods for easy identification of the nerve at the point of entry into the larynx.

## 5. Conclusion

The identification of the recurrent nerve is a must for any ENT and general surgeons. The superior approach of the recurrent laryngeal nerve is a reliable alternative. This approach is insensitive to variations induced by thyroid pathology. It first allows to reliably visualize the recurrent laryngeal nerve at the point of entry. The adaptation of this technique as a method of identifying recurrent nonspecific nerve in situations where the diagnosis is established in advance would be a source of prevention of recurrent morbidity. The knowledge of landmarks and anatomical structure neighborhood can be used to dissect in a bloodless area and avoid the nervous incident. Among the multiple benchmarks of multiple approaches to the recurrent laryngeal nerve, it is concluded that the point of entry into the larynx remains the landmark found and reliable and not modifiable by variations due to thyroid pathology.

## References

[1] Makay O., Icoz G., Yilmaz M., Akyildiz M., Yetkin E. (2008). The recurrent laryngeal nerve and the inferior thyroid artery-anatomical variations during surgery. *Langenbeck’s Archives of Surgery*.

[2] Al-Qurayshi Z., Randolph W., Alshehri M., Kandil E. (2016). Analysis of variations in the use of intraoperative nerve monitoring in thyroid surgery. *JAMA Otolaryngology–Head & Neck Surgery*.

[3] Butskiy O., Chang B. A., Luu K., McKenzie R. M., Anderson D. W. (2018). A systematic approach to the recurrent laryngeal nerve dissection at the cricothyroid junction. *Journal of Otolaryngology-Head and Neck Surgery*.

[4] Lubrano D., Levy-Chazal N., Araya Y., Avisse C. (2002). La recherche du nerf laryngé inférieur ou récurrent lors d’une lobectomie thyroïdienne. *Annales de Chirurgie*.

[5] Page C., Peltier J., Charlet L., Laude M., Strunski V. (2006). Superior approach to the inferior laryngeal nerve in thyroid surgery: anatomy, surgical technique and indications. *Surgical and Radiologic Anatomy*.

[6] Wang C. (1975). The use of the inferior cornu of the thyroid cartilage in identifying the recurrent laryngeal nerve. *Surgery, Gynecology & Obstetrics*.

[7] Koné F. I., Soumaoro S., Haïdara A. W., Cissé N., Diarra K. (2019). How to prevent recurrent morbidity during thyroidectomy for giant goiter. *Experiments in Rhinology & Otolaryngology*.

[8] Zanaret G. B., Guy L. E., Santini J. (2006). Les différents types de chirurgie. *Chirurgie de la thyroïde et de la parathyroïde*.

[9] Ngo Nyekia A. R., Njock L. R., Miloundja J., Evehe Vokwely J. E., Bengono G. (2015). Repérage préopératoire du nerf laryngé inférieur lors des thyroïdectomies. *Les Annales Françaises d’oto-rhino-laryngologie et de pathologie cervico-faciale*.

[10] Uen Y.-H., Chen T.-H., Shyu J.-F. (2006). Surgical anatomy of the recurrent laryngeal nerves and its clinical applications in Chinese adults. *Surgery Today*.

[11] Sasou S., Nakamura S. i., Kurihara H. (1998). Suspensory ligament of berry: its relationship to recurrent laryngeal nerve and anatomic examination of 24 autopsies. *Head & Neck*.

